# Closed-loop conductance scanning tunneling spectroscopy: demonstrating the equivalence to the open-loop alternative

**DOI:** 10.3762/bjnano.6.113

**Published:** 2015-05-06

**Authors:** Chris Hellenthal, Kai Sotthewes, Martin H Siekman, E Stefan Kooij, Harold J W Zandvliet

**Affiliations:** 1 Physics of Interfaces and Nanomaterials, MESA+ Institute for Nanotechnology, University of Twente, P.O. Box 217, 7500 AE, Enschede, Netherlands

**Keywords:** image charge, scanning tunneling spectroscopy (STS), tunneling barrier, work function, *z*(*V*)

## Abstract

We demonstrate the validity of using closed-loop *z*(*V*) conductance scanning tunneling spectroscopy (STS) measurements for the determination of the effective tunneling barrier by comparing them to more conventional open-loop *I*(*z*) measurements. Through the development of a numerical model, the individual contributions to the effective tunneling barrier present in these experiments, such as the work function and the presence of an image charge, are determined quantitatively. This opens up the possibility of determining tunneling barriers of both vacuum and molecular systems in an alternative and more detailed manner.

## Introduction

Although the scanning tunneling microscope (STM) has been used for the topographical imaging of conductive samples since the early 1980s [1], recent times have seen an increasing interest in the possibilities of (semi-)quantitative analysis offered by scanning tunneling spectroscopy (STS). STS measurements are typically performed in a *X*(*Y*) format, where variable *Y* is actively driven and the response of variable *X* is measured, with all other system variables being kept constant. Numerous types of STS techniques can be and have been performed on a wide variety of samples, with each different type of measurement yielding information on distinct properties of the probed sample [2].

The local density of states of a sample (LDOS) provides insight into the electronic and chemical properties of a sample. By making a spatial map of the LDOS, standing wave patterns and local electron distributions can be visualized, enabling further understanding of the exact local quantum behavior of features on the surface [3–4]. LDOS information is typically extracted through open-loop *I*(*V*) measurements, although recent studies have reported on the possibility of obtaining LDOS information by using closed-loop *z*(*V*) measurements [5–8].

Another field of interest is the determination of the work function of materials, through the use of either STS or mechanical break junction (MBJ) measurements. In the case of STS measurements, perhaps the most simple method of determining the work function is performing *I*(*z*) spectroscopy and plotting the natural logarithm of the measured tunneling conductance *G* as function of the tip–sample distance. The slope of the obtained line is equal to the inverse decay length κ which, for low bias voltages, is proportional to the square root of the work function. Several papers have also been written on the validity of applying this same method to *z*(*V*) spectroscopy measurements [9–11], although no direct comparison between *z*(*V*) and *I*(*z*) measurements was performed. Another method relies on the observation of so-called Gundlach oscillations [12–14]. These oscillations can be observed in *I*(*V*) and *z*(*V*) measurements, but require the use of bias voltages that exceed the work function of the probed sample, often necessitating bias voltages in excess of 4 V. In order to get around this restriction, recent studies have focussed on the application of transition voltage spectroscopy (TVS) [15–17]. By determining the bias voltage at which traditional tunneling is replaced by Fowler–Nordheim transport as a function of the tip–sample distance, the work function can be determined at relatively low bias voltages of about 2 V.

A potential source of error when determining the work function in a vacuum system is the presence of image charges [18–19]. These image charges are induced by tunneling electrons and have an attractive interaction with them, lowering the measured effective barrier height. If one simply assumes that the effective barrier height is equal to the work function, the presence of image charges will lead to values for 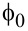 that are significantly lower than one would expect.

Determining the tunnel behavior in molecular junctions can give an indication of the properties of the molecule under consideration, and extensive research has been performed on numerous different systems [20–24]. In 2004, Engelkes et al. determined the resistance of a molecular junction as a function of the length of the used molecule [25]. Additionally, the effective mass of electrons has also been determined through the use of tunneling measurements [26]. Another active area of research deals with the interfaces between the molecule and the metal contacts making up the junction [27].

In this article, the effective tunneling barrier height is determined through the use of conductance measurements performed in *I*(*z*) and *z*(*V*) spectroscopy mode. The equivalence of both methods is demonstrated by comparing the obtained results and plotting them in a single graph. Additionally, an alternative numerical method of determining the work function of a sample through *I*(*z*) and *z*(*V*) spectroscopy is presented. Using the proposed method enables one to decouple the contributions of the work function 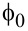 and the image charge to the effective potential barrier 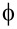. Furthermore, there is no need for the elevated bias voltages associated with Gundlach oscillations and TVS measurements. Finally, the fact that the method can be applied by using *z*(*V*) spectroscopy means that it can also be used with STM devices that can only measure in closed-loop mode.

## Model

An often used expression for the tunneling current was introduced by Simmons in 1963 [18] and is given as

[1]
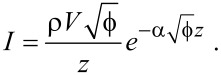


Here *I* is the tunneling current, ρ = ρ(*V*) is the energy-dependent combined density of states of the tip and the sample, *V* is the applied tip–sample bias voltage, *z* is the tip–sample distance, 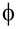 is the tunneling barrier and 
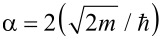
, with *m* the rest mass of the electron. The product 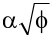 is sometimes referred to as the inverse decay length

[2]
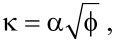


and provides a measure of the change in tunneling current for a given decrease or increase of the tip–sample separation.

Assuming a symmetrical, rectangular barrier, the term 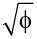 is simply equal to the square root of the combined vacuum work function of the tip–sample system, i.e.,

[3]
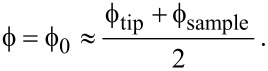


However, applying a bias voltage between the tip and sample causes the barrier to lower in an asymmetrical fashion:

[4]
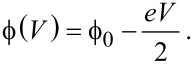


Any charge travelling between the tip and the sample will induce an image charge of equal magnitude but opposite polarity. In addition to lowering the barrier, the presence of an image charge effect will also narrow it. This effect can be included in the Simmons model by replacing the tip–sample separation *z* with the effective barrier width *s*:

[5]
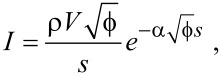


where the effective barrier width *s* is given by [18,28]:

[6]
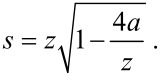


Here, *a* is given by

[7]
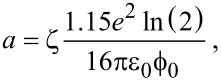


with ε_0_ the electric permittivity of the vacuum and ζ a constant between 0 (two point charges) or 1 (two infinite parallel plates) determining the strength of the image charge effect.

The lowering effect of the image charge can be included in the effective barrier expression as follows [18]:

[8]
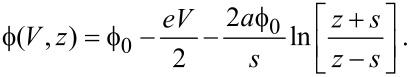


Note that for small values of *V* and ζ = 0, Equation 8 reduces to Equation 3 and Equation 5 reduces to Equation 1.

### Current–distance spectroscopy

An often used and reasonably accurate way of determining 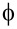 from *I*(*z*) measurements consists of plotting the natural logarithm of the conductance *G* of the tunneling barrier as a function of *z*. The conductance is equal to the measured tunneling current divided by the tip–sample bias, i.e., *G* = (*I*/*V*). The linear slope of the obtained line is then approximately equal to κ, as can be deduced from Equation 1 and Equation 2. In the absence of image charge effects, the work function 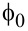 can then be obtained through Equation 4.

The same method can be used when including the effect of image charges, although the inverse decay length will take a slightly different form:

[9]
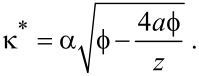


It is directly apparent that a significant image charge effect will have a bending effect on the ln(*G*)-vs-*z* curve. However, a quantitative analysis is made difficult by the non-straightforward dependence of κ^*^ on ζ.

In order to make a quantitative analysis possible, a numerical method will have to be developed. As a starting point, Equation 5 will have to be rewritten to eliminate as many unknown parameters as possible. By taking the the derivative *dI*/*dz*, the density of states ρ can be eliminated from the equation as follows:

[10]
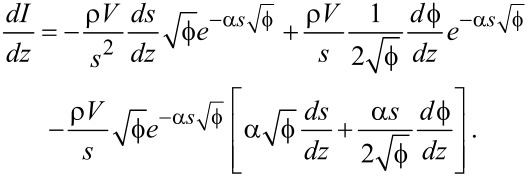


Substituting Equation 5 then gives

[11]
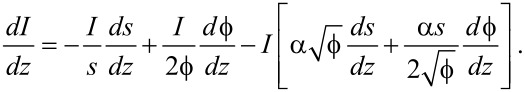


Working through all the partial derivatives (see Supporting Information File 1 for a full derivation) eventually yields

[12]
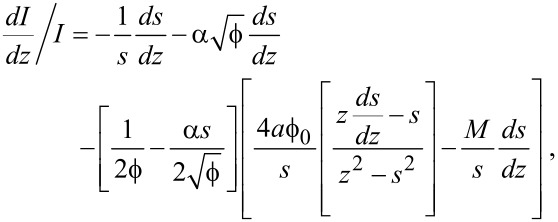


with

[13]
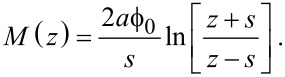


This resulting equation can be used to determine the work function 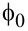 and image charge constant ζ from a standard, open-loop *I*(*z*) measurement, as they are the only unknown variables remaining. These two unknowns can be extracted from measured data by fitting Equation 12 to the measured *I*(*z*) data in a least-squares fashion.

### Constant-current spectroscopy

When it is not possible, or not desirable, to perform experiments in open-loop mode, the effective barrier can also be determined from closed-loop *z*(*V*) experiments. As is the case for *I*(*z*) measurements, plotting the natural logarithm of the conductance as a function of tip–sample separation yields a good first approximation of the inverse decay length [10–11]. However, quantitatively decoupling the contributions of 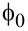 and ζ once again requires the use of a derivative numerical method. Taking into account that *I* does not vary as a function of *V* during closed-loop experiments, Equation 5 can be rewritten as:

[14]
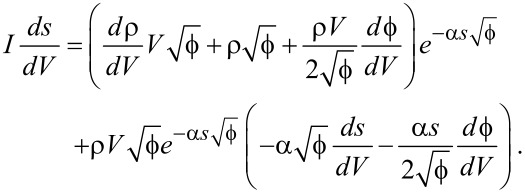


Substituting Equation 5 into this expression and dividing by *I* gives:

[15]
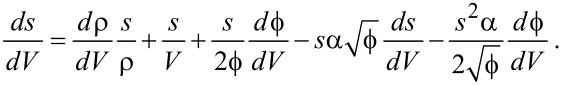


Following the derivation given in Supporting Information File 1, this leads to the full expression

[16]
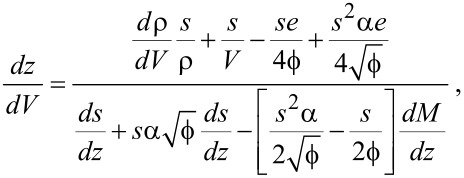


with *M* given by Equation 13.

By using a least-squares fitting routine with parameters 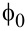 and ζ, one can fit Equation 16 to the *dz*/*dV* data obtained from the experiment and, as such, determine the work function and image charge constant of the system.

An important limitation to the use of *z*(*V*) measurements for the determination of the effective barrier height arises due to the density of states (DOS) of the studied sample. For samples with a featureless or weakly varying DOS, measured at limited bias voltages, the (*dρ*/*dV*)(*s*/ρ) term in Equation 16 can be neglected. However, for non-featureless densities of states, the LDOS of the tip and sample will have to be known in order to fully evaluate Equation 16. This necessitates additional or consecutive measurements in order to obtain the system LDOS [5–8,29].

## Results and Discussion

**Figure 1:**
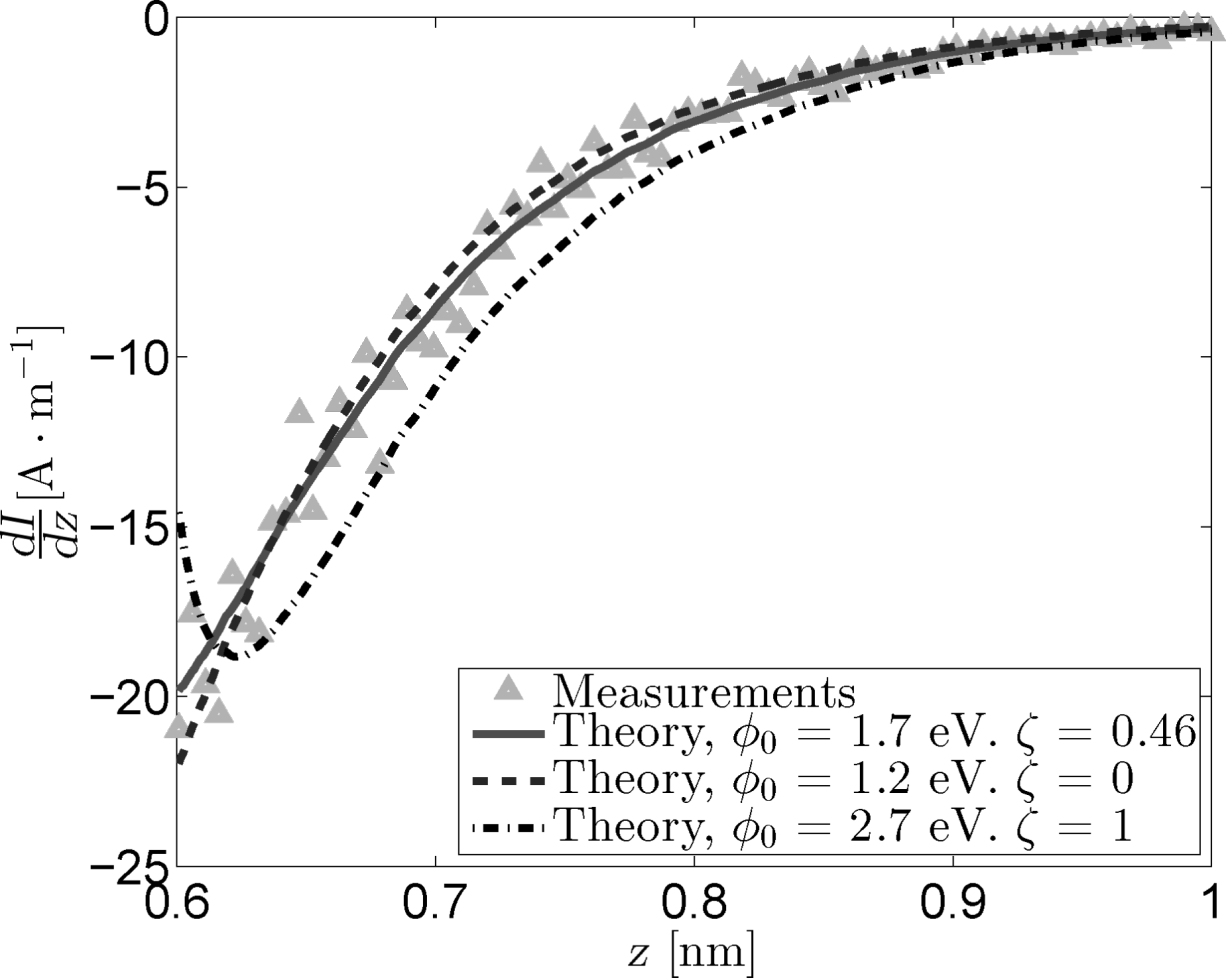
Measured and reconstructed *dI*/*dz* data obtained from *I*(*z*) measurements using three sets of fit parameters. The results for the “free” fit and the fit in absence of an image charge strongly resemble one another, indicating a masking interaction between 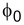 and ζ.

To determine the effect of ζ on 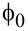, three different sets of fit parameters were used: no image charge (ζ = 0), maximum image charge (ζ = 1) and variable image charge. The *dI*/*dz* signal determined from the measurement and the traces that were reconstructed from the fitted parameters can be seen in Figure 1.

The most obvious conclusion that can be drawn from the obtained values of 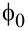 and ζ is that the two are coupled, with an increase in one leading to an increase in the other. While there is a difference of 0.5 eV between 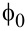 for the optimal fit and 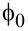 in absence of an image charge, this seems to have little to no effect on the reconstructed signal. This shows that it is quite difficult to discriminate between the contributions of the effective work function and the image charge, as a change in one variable can be readily masked by a change in the other. The reconstructed trace for ζ = 1, however, shows that there is a limit to this masking effect. For higher values of ζ, 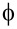 becomes more strongly dependent on *z*, which manifests itself in a change in curvature for the *dI*/*dz* signal.

**Figure 2:**
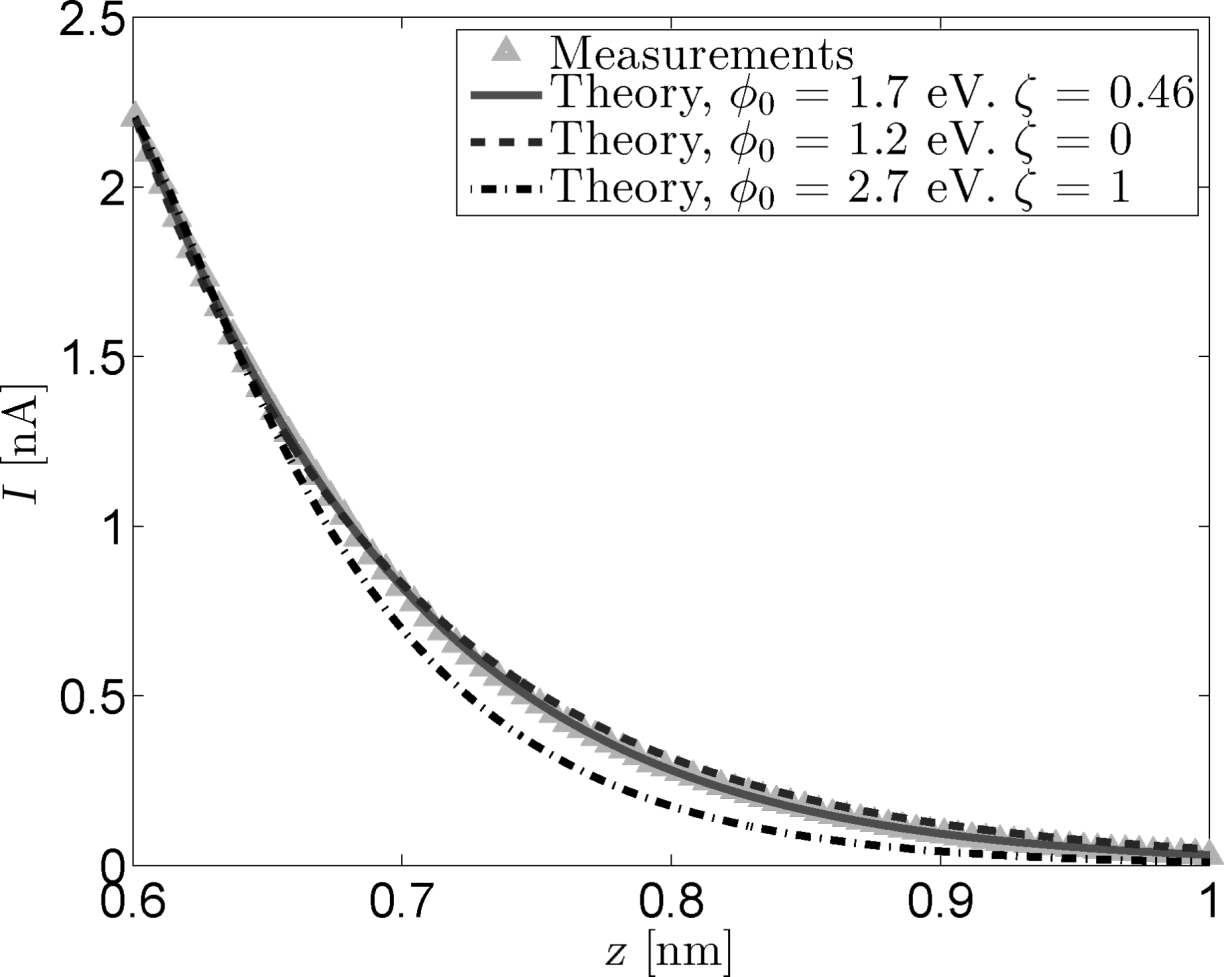
Measured and reconstructed *I*(*z*) data. The reconstructed traces were based on the fit parameters from Figure 1. All curves have been normalized to have a starting point of 2.2 nA at *z*_0_ = 0.6 nm.

Figure 2 shows the current signals reconstructed from the fit parameters shown in Figure 1 alongside the measured *I*(*z*) curve. All curves have been normalized to have the same starting point of roughly 2.2 nA at *z*_0_ = 0.6 nm. This value for *z*_0_ was estimated based on previous STM measurements [17] and will be used for all following analysis. While there is always a certain margin of error in estimating *z*_0_, changing this value by a few angstroms does not significantly impact the results of the analysis, as can be seen in Supporting Information File 1. Figure 2 further emphasizes the difficulty in discriminating between the measured signal, the optimal fit reconstruction, and the reconstruction that does not incorporate image charge effects. The image also shows the decrease in curvature for the ζ = 1 trace, although the difference between this trace and the measured signal is still rather small.

**Figure 3:**
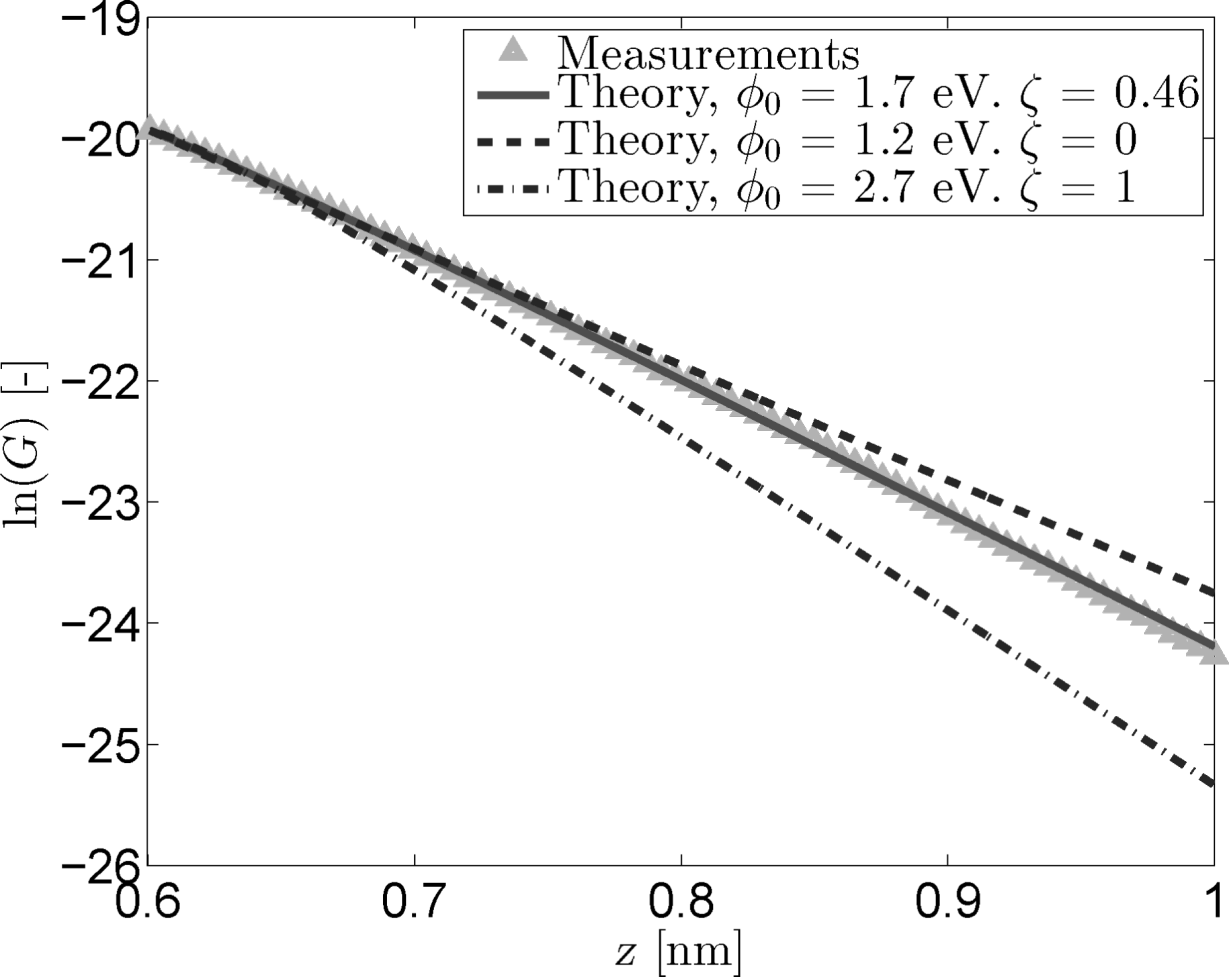
Logarithmic conductance versus tip-sample distance for measured and reconstructed *I*(*z*) data. The reconstructed traces were based on the fit parameters from Figure 1. The inclusion of an image charge term gives the reconstructed trace the curvature needed to follow the measured data.

In order to further elucidate the effect of the image charge on the tunneling current, the logarithm of the measured conductance and the conductance reconstructed from the fitted parameters has been plotted in Figure 3 as a function of *z*. As mentioned in the Model section, the slope of these traces is equal to the inverse decay length κ^*^. Figure 3 clearly shows the effect of including the image charge when reconstructing the tunneling current. While the effect is negligible for small tip–sample separations, the inclusion of an image charge term introduces a clear deviation from the linear trend observed for the reconstructed non-image charge current at larger separations. This same non-linearity is observed in the logarithm of the measured conductance, proving the need for the inclusion of a *z* dependent term in 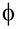.

**Figure 4:**
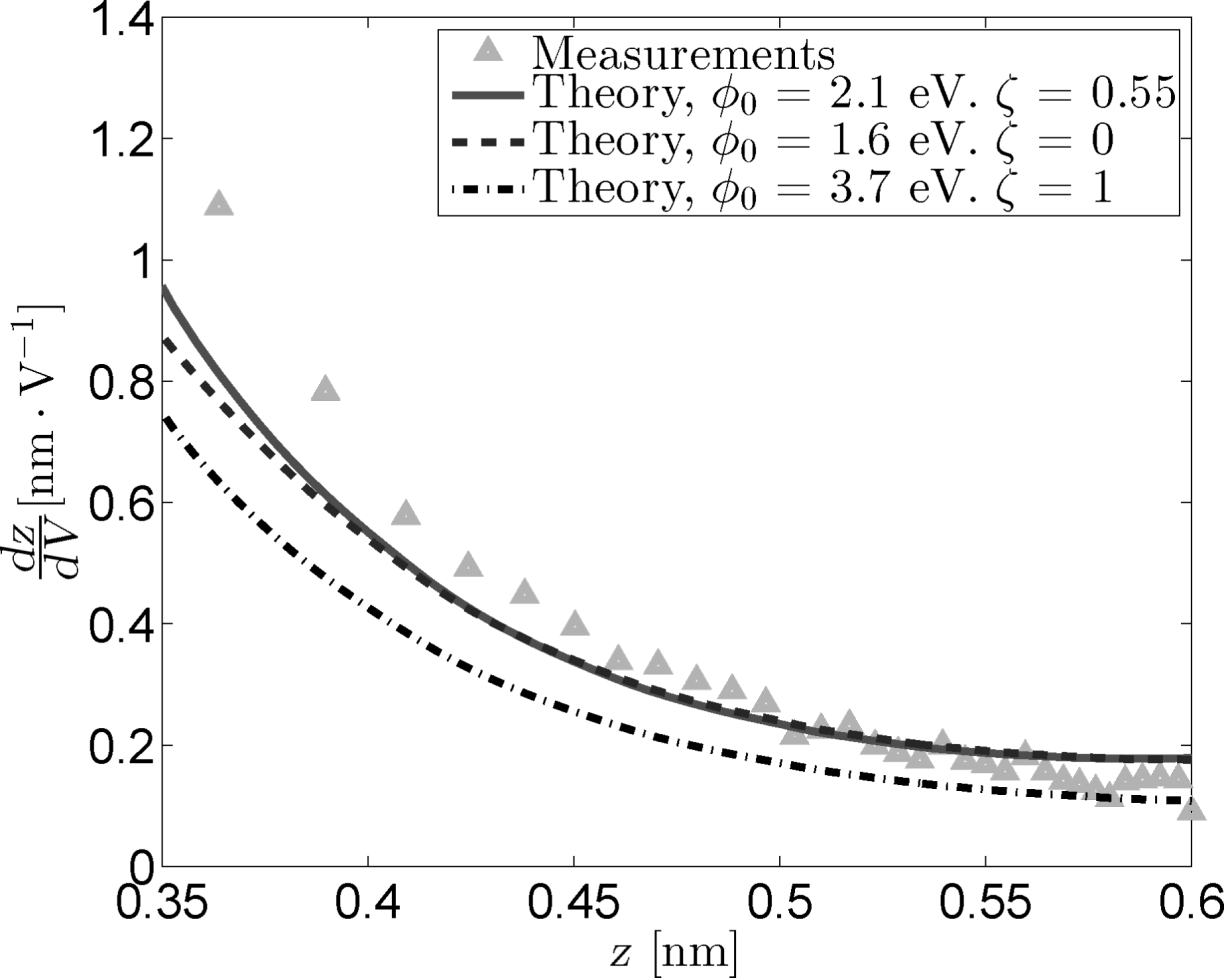
Measured and reconstructed *dz*/*dV* traces obtained from *z*(*V*) measurements by using three sets of fit parameters. As is the case for the *I*(*z*) data, the “free” fit and the fit in absence of an image charge overlap nearly completely.

Figure 4 shows the measured and reconstructed *dz*/*dV* traces. The reconstructed traces were based on Equation 16 under the assumption that the LDOS is slowly varying within the probed bias range. The difference between the optimal fit parameters and those obtained through the fit excluding image charge effects is very similar to that observed for the *I*(*z*) measurements. In addition, the difference between the reconstructed traces is practically invisible due to the masking effect of 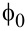 on lower values of ζ. Forcing a maximum image charge contribution leads to an increase of 1.6 eV in 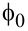, but also decreases the quality of the fit.

**Figure 5:**
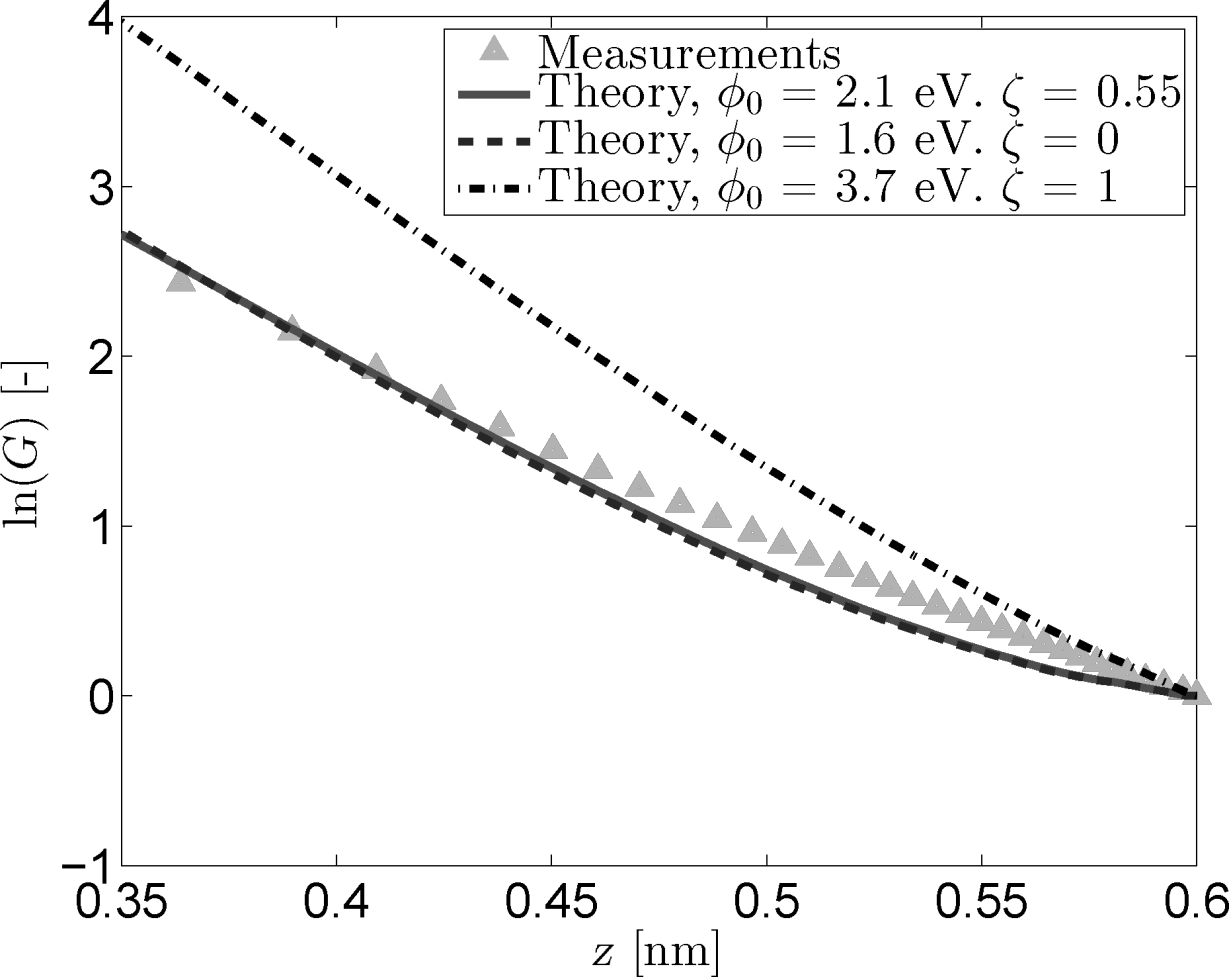
Logarithmic conductance versus tip-sample distance for measured and reconstructed *z*(*V*) data. The reconstructed traces were based on the fit parameters from Figure 4. The logarithmic offsets have been subtracted so that ln(*G*) = 0 at *z*_0_ = 0.6 nm.

The conductance plots shown in Figure 5 were obtained in the same manner as the *I*(*z*) conductance plots with the important difference that the offsets were subtracted to ensure that all curves start at *z*_0_ = 0.6 nm. While the reconstructed *I*(*z*) curves can be scaled to have the same setpoint current, this same method can not be applied to the *z*(*V*) data. Instead, Equation 5 is used to reconstruct the (constant) current by using ρ = 1. This does not influence the slope of the obtained conductance curves, but it will introduce different offsets for each curve, which is why it is necessary to zero them on a common point. The conductance plot obtained in this manner (Figure 5) reveals a prominent curvature around *z* = 0.55 nm for the fitted parameters that is not present in the measurements. Additionally, the optimal fit and the fit excluding image charges perfectly overlap, further demonstrating the masking effect of 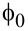 and ζ. The overall quality of the fitted traces is lower than that of the fitted *I*(*z*) traces. This could indicate an additional or stronger dependence of the tunneling current on the applied bias voltage or tip–sample separation than is present in the used model.

**Figure 6:**
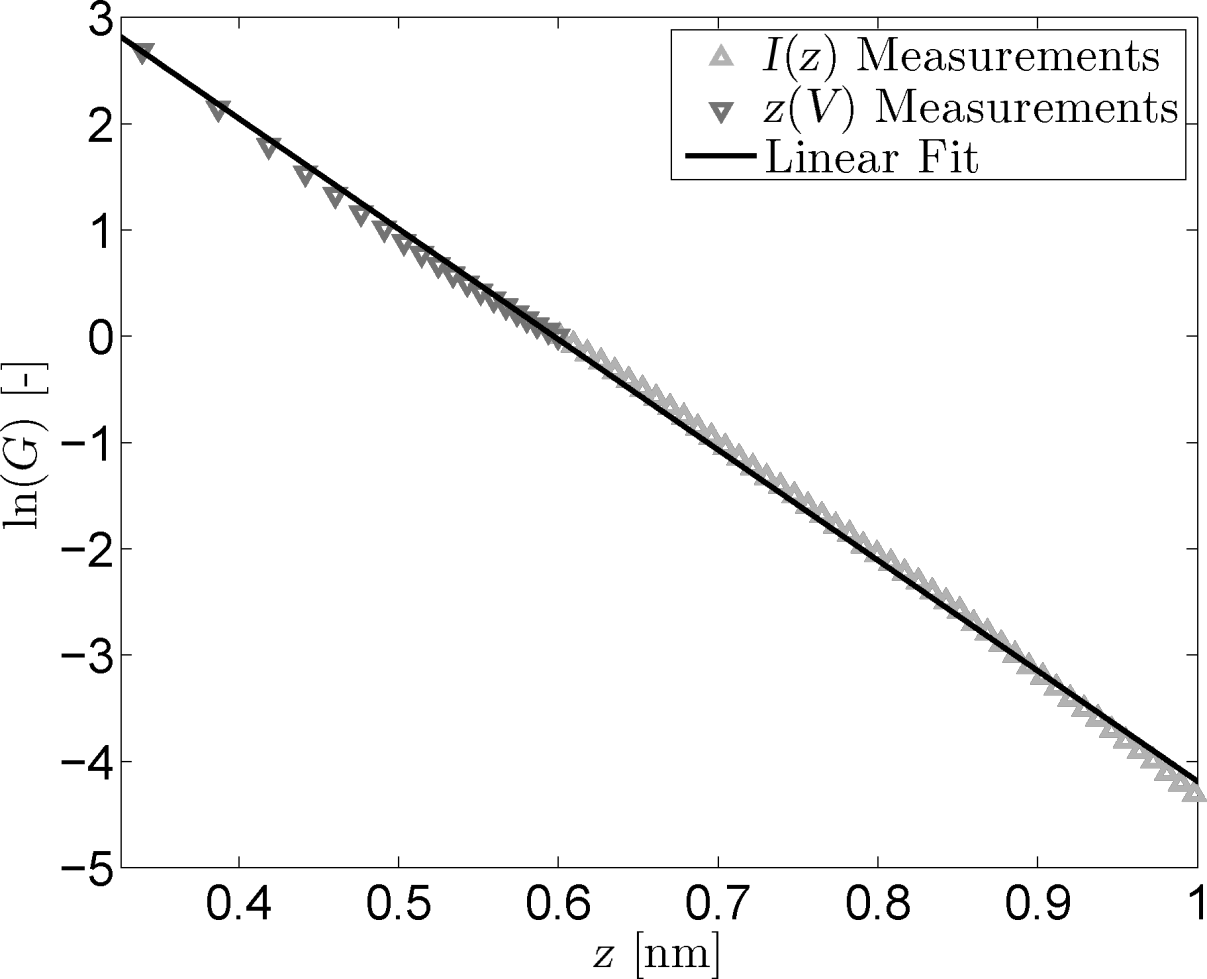
Combined logarithmic conductance versus tip–sample distance for *z*(*V*) and *I*(*z*) measurements. Logarithmic offsets for both types of measurements were zeroed at *z*_0_ = 0.6 nm. The linear fit has a slope of −10.4 nm^−1^.

By plotting the results of both *I*(*z*) and *z*(*V*) measurements in one figure, the equivalence of both methods can be demonstrated. Figure 6 shows the conductance data obtained from the *I*(*z*) and *z*(*V*) measurements, alongside a linear fit. The offsets of both data sets have been removed to ensure their intersection at *z* = 0.6 nm. From this figure, it is clear that the different types of measurements blend together practically seamlessly and that their slopes match closely. The fitted slope is equal to −10.4 nm^−1^; approximately twice as small as the theoretical value. This leads to an effective barrier of 1 eV; approximately four to five times as small as the theoretical value [30–31]. Measurements performed with different W tips and/or different Au(111) samples yielded similar barrier heights.

Despite the fact that the conductance plots of the *I*(*z*) and *z*(*V*) measurements demonstrate that both methods are equally viable for the determination of the effective tunneling barrier, the obtained values for 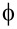 and 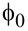 are lower than they should be according to theory. Additionally, the fitted parameters for the *z*(*V*) and *I*(*z*) measurements are not fully consistent and the fits themselves are not as accurate as one would hope. Abnormally low barriers obtained through STS studies have been reported in the past, with a variety of possible reasons being suggested [26,32–37].

As early as 1982, Binnig et al. reported work functions below 1 eV for a tungsten–platinum system, which they ascribed to poor vacuum conditions and contamination [32]. Similarly, the presence of water layers in the tunneling junction can also lead to lower apparent barrier heights [35,38]. However, the measurements presented in this article have been performed under UHV conditions, making it unlikely that (water) contamination is the cause of the low apparent barrier heights extracted from them.

Erroneous barrier heights can also be caused by misinterpretation of the tip–sample separation due to relaxation effects [36,39–40]. According to experimental results and theoretical calculations, these effects only take place at tip–sample separations below 500 pm, i.e., in the *z*(*V*) regime of Figure 6. As such, any relaxation effects will be negated by the active feedback loop during *z*(*V*) measurements. If this were not the case, the onset of relaxation effects below 500 pm should lead to a change in the slope of the measurements presented in Figure 6. Following the same line of reasoning, it is implausible that short-range electrostatic and exchange-interactions as suggested by Lang [34] are responsible for the low extracted barrier values.

In order to analyze direct tunneling experiments on large-area molecular junctions, Akkerman et al. included an additional exponential scaling factor in the Simmons model, which they later ascribed to the effective mass of the electrons tunneling through the molecules [26]. A similar effective mass correction has also been applied to tunneling junctions consisting of a single molecule attached to both the STM tip and the sample and to tip-molecule-vacuum-sample junctions [27,41–42]. While a non-unity effective mass does indeed lead to lower apparent barrier heights, the tip–vacuum–sample system described in this article should not contain any elements that could lead to the emergence of such an effective mass.

By introducing an additional scaling factor γ in the Simmons model, analogous to Akkerman et al. [26], the performance of the fitting model can be assessed for data values that result in a barrier height that is closer to theoretical values. Equation 5 is rewritten in the following form:

[17]
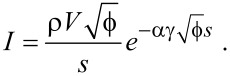


**Figure 7:**
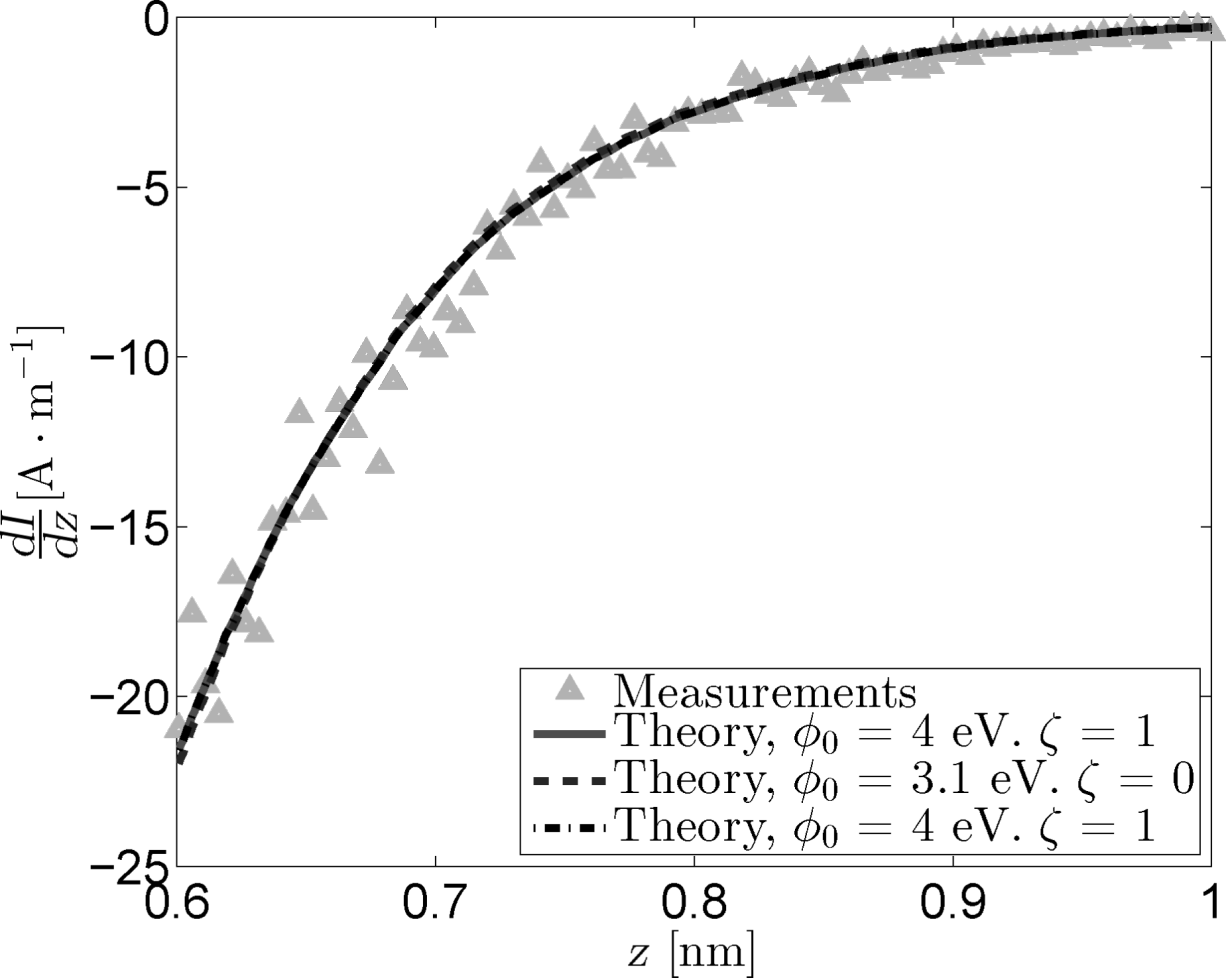
Measured and reconstructed *dI*/*dz* data obtained from *I*(*z*) measurements for γ = 0.5. The masking effect between 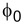
and ζ remains, but changes in these parameters do not significantly impact the effective barrier, leading to all reconstructed traces overlapping. The free parameter fit (solid line) leads to the maximum value for ξ (i.e., ξ = 1), causing the fitted parameters to be identical to those obtained from the fit with ξ fixed at its maximum value (dashdotted line).

**Figure 8:**
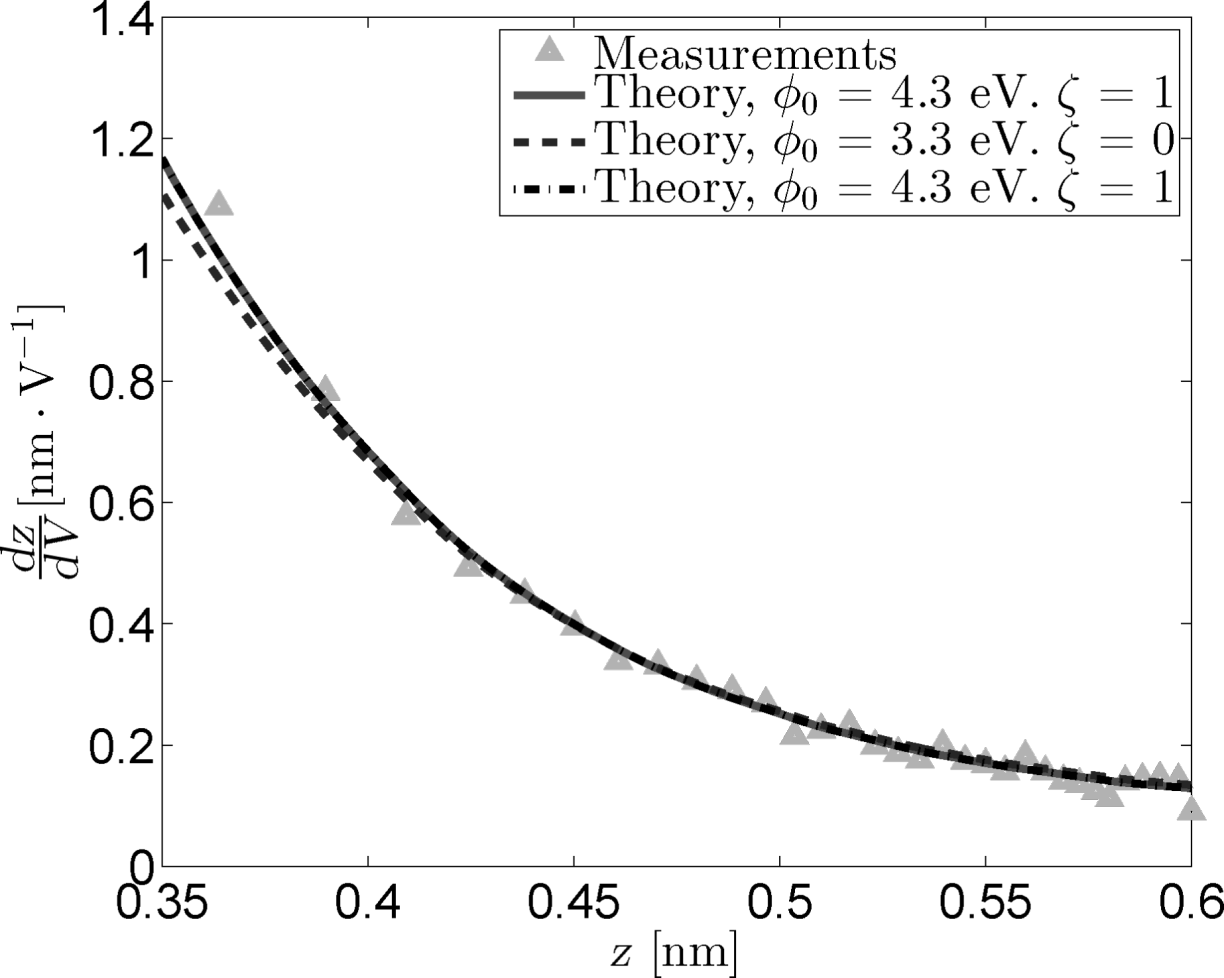
Measured and reconstructed *dz*/*dV* traces obtained from *z*(*V*) measurements for *γ=0.5*. The masking effect between 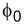 and ζ remains, but changes in these parameters do not significantly impact the effective barrier, leading to all reconstructed traces overlapping. The free parameter fit (solid line) leads to the maximum value for ξ (i.e., ξ = 1), causing the fitted parameters to be identical to those obtained from the fit with ξ fixed at its maximum value (dashdotted line).

Based on the linear slope extracted from Figure 6, γ was chosen to be equal to 0.5. As can be seen in Figure 7 and Figure 8, the introduction of γ has three major consequences. Firstly, the quality of both fits increases dramatically, especially for the *z*(*V*) data. Secondly, the obtained values for 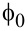 and ζ are now consistent between measurements, with only a 0.3 eV difference in 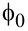 between the *I*(*z*) and *z*(*V*) measurements. Finally, the influence of the different fitted parameters on the shape of the reconstructed curves has diminished sharply, with all three curves overlapping for both types of measurements. In other words, while the mutual masking effect between 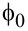 and ζ remains, the effective barrier 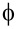 is largely unaffected by changes in these parameters. This is consistent with a theoretical analysis performed by Coombs et al. [28], which showed that the effect of an image charge on the apparent tunneling barrier height is not readily extracted from *I*(*z*) data. While the exact origin of γ is unclear, it is undeniable that its inclusion increases both the quality of the fits and the accuracy of the obtained values.

## Conclusion

The conductances obtained from *I*(*z*) and *z*(*V*) spectroscopy can both be used to get a good indication of the effective tunneling barrier height 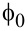 when plotted logarithmically as a function of the tip–sample separation. This means that the determination of the (local) work function of a material can be performed purely in the closed-loop operation mode present in all STM systems. Numerical analysis reveals a strong masking interaction between 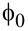 and ζ, showing that neglecting the presence of an image charge can lead to one underestimating the value of 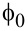.

The inclusion of an additional scaling factor γ has a positive impact on both the quality of the numerical fits as well as the values obtained from them. The exact physical origin of this term is, as of yet, unknown.

## Experimental

Experiments were performed on an RHK ultra-high vacuum system at room temperature and a base pressure of 10^−10^ mbar. Measurement data was collected on a hydrogen flame-annealed Au(111) sample by using an electrochemically etched W tip. The sample and tip had both been exposed to ultra-high vacuum conditions for several weeks prior to measuring. Spectroscopy traces were acquired by using an RHK IVP-200 preamplifier at a fixed gain of 10^8^ V·A^−1^ and were performed within a short timeframe to minimize the effects of drift and possible changes to the tip or sample.

Experimental parameters were chosen to prevent changes in tip–sample distance between experiments. As such, *z*(*V*) measurements were performed at a setpoint current of 2 nA over a range of 1 to 0.05 V. Subsequently, *I*(*z*) measurements were performed at a current setpoint of 2 nA and a bias setpoint of 1 V. The *I*(*z*) and *z*(*V*) measurements presented in this article are based on an average of 200 traces per measurement. A topography image of the sample can be found in Supporting Information File 1.

## Supporting Information

File 1 Additional experimental data.
