# Supplementary material for: Closed-loop conductance scanning tunneling spectroscopy: demonstrating the equivalence to the open-loop alternative
Source: Beilstein J Nanotechnol. 2015 May 6;6:1116–24. doi: 10.3762/bjnano.6.113 (PMC4464298; doi:10.3762/bjnano.6.113)
Supplement: File 1 — Additional experimental data. [file Beilstein_J_Nanotechnol-06-1116-s001.pdf]

Supporting Information

for

Closed-loop conductance scanning tunneling spectroscopy: demonstrating the equivalence to the open-loop alternative

Chris Hellenthal, Kai Sotthewes, Martin H. Siekman, E. Stefan Kooij* and Harold J. W. Zandvliet

Address: Physics of Interfaces and Nanomaterials, MESA+ Institute for Nanotechnology, University of Twente, P.O. Box 217, 7500 AE, Enschede, Netherlands

Email: E. Stefan Kooij - e.s.kooij@utwente.nl

* Corresponding author

Additional experimental data

Raw data and fit dependence

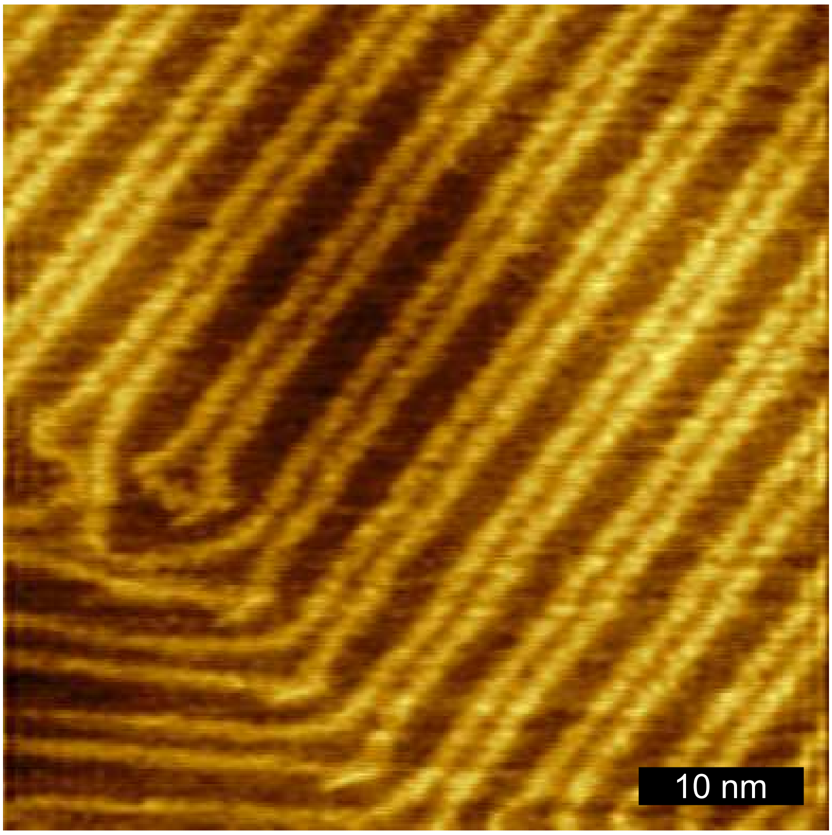

Figure S1: 50 × 50 nm STM topography image of the Au(111) surface. The herringbone recon-struction is clearly visible.

Figure S1 shows a topography image of the Au(111) surface on which the measurements presented in the main article were performed. The herringbone reconstruction that is typical for the Au(111) surface is clearly visible, indicating good tip/sample conditions.

Figure S2 shows a number of raw data traces for the $I(z)$ and $z(V)$ spectroscopy measurements. Offsets have been added to facilitate distinguishing individual traces. The traces analyzed in the main article were obtained by averaging approximately 200 raw data traces per measurement.

The influence of z_0 on the outcome of the fitting routine is shown in Figure S3. It should be noted that for low values of z_0 the $z(V)$ measurements at $\zeta = 1$ yield complex (non-physical) results due to the effective barrier becoming negative.

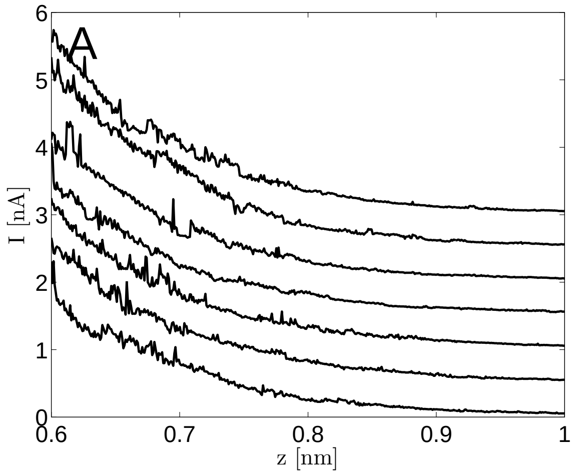

Figure S2: Raw spectroscopy traces. a) $I(z)$ spectroscopy. b) $z(V)$ spectroscopy. Offsets have been added to individual traces to facilitate analysis.

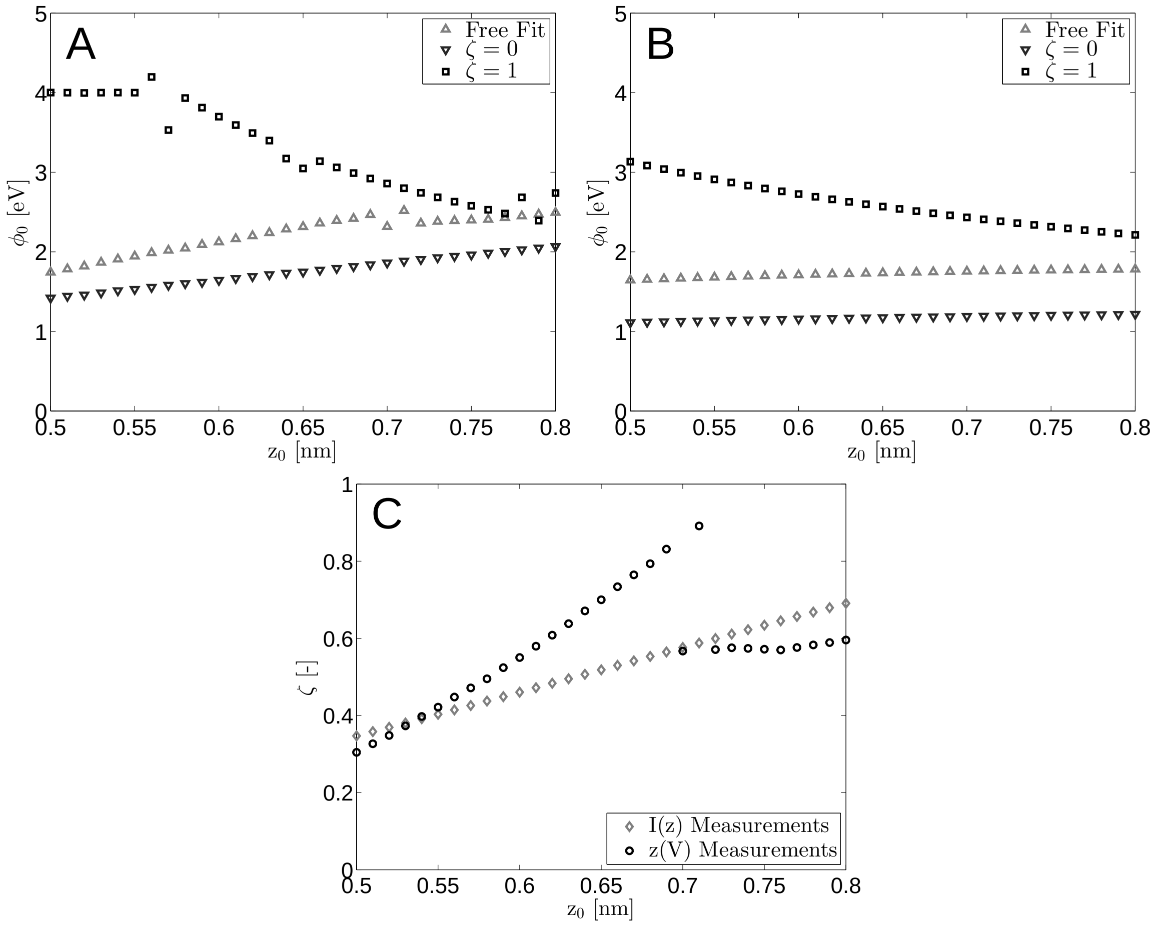

Figure S3: Fit parameter dependence on z_0. a) ϕ_0 dependence for z(V) measurements. b) ϕ_0 dependence for I(z) measurements. c) ζ dependence for both types of measurement.

Model derivation

Current–distance spectroscopy

As a starting point, the image charge corrected current equation

$$I = \frac{\rho V \sqrt{\phi}}{s} e^{-\alpha\sqrt{\phi}s}, \tag{1}$$

will have to be rewritten to eliminate as many unknown parameters as possible. By taking the derivative $\frac{dI}{dz}$, the density of states ρ can be eliminated from the equation as follows:

$$\begin{aligned} \frac{dI}{dz} = &-\frac{\rho V}{s^2}\frac{ds}{dz}\sqrt{\phi}e^{-\alpha s\sqrt{\phi}} + \frac{\rho V}{s}\frac{1}{2\sqrt{\phi}}\frac{d\phi}{dz}e^{-\alpha s\sqrt{\phi}} \\ &-\frac{\rho V}{s}\sqrt{\phi}e^{-\alpha s\sqrt{\phi}}\left[\alpha\sqrt{\phi}\frac{ds}{dz} + \frac{\alpha s}{2\sqrt{\phi}}\frac{d\phi}{dz}\right]. \end{aligned} \tag{2}$$

Substituting Equation 1 then gives

$$\frac{dI}{dz} = -\frac{I}{s}\frac{ds}{dz} + \frac{I}{2\phi}\frac{d\phi}{dz} - I\left[\alpha\sqrt{\phi}\frac{ds}{dz} + \frac{\alpha s}{2\sqrt{\phi}}\frac{d\phi}{dz}\right]. \tag{3}$$

For the sake of brevity, the image charge term can be redefined as

$$M(z) = \frac{2a\phi_0}{s}\ln\left[\frac{z+s}{z-s}\right], \tag{4}$$

which leads to

$$\frac{d\phi}{dz} = -\frac{dM}{dz}. \tag{5}$$

This derivative can be determined by using the product rule:

$$\frac{dM}{dz} = \ln\left[\frac{z+s}{z-s}\right]\frac{d}{dz}\frac{2a\phi_0}{s} + \frac{2a\phi_0}{s}\frac{d}{dz}\ln\left[\frac{z+s}{z-s}\right]. \tag{6}$$

The first part of this equation then yields

$$\ln\left[\frac{z+s}{z-s}\right]\frac{d}{dz}\frac{2a\phi_0}{s} = -\frac{2a\phi_0}{s^2}\frac{ds}{dz}\ln\left[\frac{z+s}{z-s}\right], \tag{7}$$

while the second part gives

$$\begin{aligned} &\frac{2a\phi_0}{s}\frac{d}{dz}\ln\left[\frac{z+s}{z-s}\right] \\ &= \frac{2a\phi_0}{s}\left[\frac{z-s}{z+s}\right]\left[\frac{1+\frac{ds}{dz}}{z-s} - \frac{(z+s)\left(1-\frac{ds}{dz}\right)}{(z-s)^2}\right] \\ &= \frac{2a\phi_0}{s}\left[\frac{1+\frac{ds}{dz}}{z+s} - \frac{1-\frac{ds}{dz}}{z-s}\right] = \frac{4a\phi_0}{s}\left[\frac{z\frac{ds}{dz}-s}{z^2-s^2}\right]. \end{aligned} \tag{8}$$

Equation 4 can be substituted into Equation 7 for further simplification, after which combination with Equation 8 finally yields

$$\frac{dM}{dz} = \frac{4a\phi_0}{s}\left[\frac{z\frac{ds}{dz}-s}{z^2-s^2}\right] - \frac{M}{s}\frac{ds}{dz}. \tag{9}$$

Inserting everything back into Equation 3 and dividing by I then gives the final expression

$$\begin{aligned} \frac{dI}{dz}/I = &-\frac{1}{s}\frac{ds}{dz} - \alpha\sqrt{\phi}\frac{ds}{dz} \\ &-\left[\frac{1}{2\phi} - \frac{\alpha s}{2\sqrt{\phi}}\right]\left[\frac{4a\phi_0}{s}\left[\frac{z\frac{ds}{dz}-s}{z^2-s^2}\right] - \frac{M}{s}\frac{ds}{dz}\right] \end{aligned} \tag{10}$$

Constant-current spectroscopy

Once again, the first step in the derivation is rewriting Equation 1:

$$I\frac{ds}{dV} = \left(\frac{d\rho}{dV}V\sqrt{\phi}+\rho\sqrt{\phi}+\frac{\rho V}{2\sqrt{\phi}}\frac{d\phi}{dV}\right)e^{-\alpha s\sqrt{\phi}}$$
$$+\rho V\sqrt{\phi}e^{-\alpha s\sqrt{\phi}}\left(-\alpha\sqrt{\phi}\frac{ds}{dV}-\frac{\alpha s}{2\sqrt{\phi}}\frac{d\phi}{dV}\right) \tag{11}$$

Substituting Equation 1 into this expression and dividing by I gives:

$$\frac{ds}{dV} = \frac{d\rho}{dV}\frac{s}{\rho}+\frac{s}{V}+\frac{s}{2\phi}\frac{d\phi}{dV}-s\alpha\sqrt{\phi}\frac{ds}{dV}-\frac{s^2\alpha}{2\sqrt{\phi}}\frac{d\phi}{dV} \tag{12}$$

Applying the chain rule, i.e.

$$\frac{ds}{dV} = \frac{ds}{dz}\frac{dz}{dV},$$

then leads to

$$\frac{ds}{dz}\frac{dz}{dV} = \frac{d\rho}{dV}\frac{s}{\rho}+\frac{s}{V}+\frac{s}{2\phi}\frac{d\phi}{dV}-s\alpha\sqrt{\phi}\frac{ds}{dz}\frac{dz}{dV}-\frac{s^2\alpha}{2\sqrt{\phi}}\frac{d\phi}{dV}. \tag{13}$$

When not including the image charge effect, $s = z$ and $\frac{ds}{dz} = 1$ and the above equation reduces to:

$$\frac{dz}{dV} = \frac{d\rho}{dV}\frac{z}{\rho}+\frac{z}{V}+\frac{z}{2\phi}\frac{d\phi}{dV}-z\alpha\sqrt{\phi}\frac{dz}{dV}-\frac{z^2\alpha}{2\sqrt{\phi}}\frac{d\phi}{dV}. \tag{14}$$

When including neither the asymmetrical lowering of the barrier due to the applied bias voltage nor the lowering/narrowing effect of an image charge $\frac{d\phi}{dV} = 0$ and Equation 14 can be rewritten as:

$$\frac{dz}{dV} = \frac{\frac{d\rho}{dV}\frac{z}{\rho}+\frac{z}{V}}{1+z\alpha\sqrt{\phi}}. \tag{15}$$

Including the asymmetric lowering of the barrier leads to $\frac{d\phi}{dV} = -\frac{e}{2}$ and

$$\frac{dz}{dV} = \frac{\frac{d\rho}{dV}\frac{z}{\rho} + \frac{z}{V} - \frac{ze}{4\phi} + \frac{z^2\alpha e}{4\sqrt{\phi}}}{1 + z\alpha\sqrt{\phi}}. \tag{16}$$

Determining the derivative of the image charge term is slighty more involved. By once again defining

$$M(z(V)) = \frac{2a\phi_0}{s}\ln\left[\frac{z+s}{z-s}\right] \tag{17}$$

the notation can be kept relatively compact. Because M only has an implicit dependence on V *via* z, the chain rule can be used once again:

$$\frac{dM}{dV} = \frac{dM}{dz}\frac{dz}{dV}, \tag{18}$$

leading to

$$\frac{d\phi}{dV} = -\frac{e}{2} - \frac{dM}{dz}\frac{dz}{dV}. \tag{19}$$

Inserting Equation 19 into Equation 13 gives the full expression

$$\frac{dz}{dV} = \frac{\frac{d\rho}{dV}\frac{s}{\rho} + \frac{s}{V} - \frac{se}{4\phi} + \frac{s^2\alpha e}{4\sqrt{\phi}}}{\frac{ds}{dz} + s\alpha\sqrt{\phi}\frac{ds}{dz} - \left[\frac{s^2\alpha}{2\sqrt{\phi}} - \frac{s}{2\phi}\right]\frac{dM}{dz}}, \tag{20}$$

with $\frac{dM}{dz}$ given by Equation 9.
